# *Escherichia coli‐* Associated Infectious Aortitis Complicated by Abdominal Aortic Aneurysm and Diverticulitis Treated With Covered Endovascular Reconstruction of the Aortic Bifurcation

**DOI:** 10.1002/ccr3.72286

**Published:** 2026-03-19

**Authors:** Ching‐Chou Pai, Ya‐Ting Chuang, Hua‐Yu Lin, Tao‐An Chen

**Affiliations:** ^1^ Department of Cardiovascular Surgery Show Chwan Memorial Hospital Changhua Taiwan; ^2^ Surgical Intensive Care Unit, Department of Nursing Show Chwan Memorial Hospital Changhua Taiwan; ^3^ Division of Respiratory Therapy, Department of Chest Medicine Show Chwan Memorial Hospital Changhua Taiwan; ^4^ Institute of Emergency and Critical Care Medicine National Yang Ming Chiao Tung University Taipei City Taiwan

**Keywords:** abdominal, aortic aneurysm, aortitis, bacteremia, endovascular procedures, *Escherichia coli*, mycotic aneurysm

## Abstract

Infectious aortitis caused by *Escherichia coli* is exceedingly rare and associated with high mortality. We report a 76‐year‐old man presenting with nonspecific abdominal symptoms and *E. coli* bacteremia, complicated by an infrarenal eccentric saccular abdominal aortic aneurysm and diverticulitis. The patient was successfully treated with percutaneous endovascular aneurysm repair (PEVAR) using a covered endovascular reconstruction of the aortic bifurcation (CERAB) technique, followed by prolonged targeted antimicrobial therapy. Early contrast‐enhanced imaging and prompt endovascular intervention may prevent catastrophic complications and improve outcomes in high‐risk patients.

## Key Clinical Message

In elderly patients with *Escherichia coli* bacteremia and nonspecific abdominal symptoms, early contrast‐enhanced imaging is essential to exclude infectious aortic aneurysm. Prompt preoperative broad‐spectrum antibiotics combined with timely endovascular repair and prolonged targeted therapy may prevent aneurysmal rupture and improve survival.

## 1 Introduction

**Infectious aortitis** is a rare but highly lethal vascular infection. Its pathophysiology primarily involves bacterial seeding of the aortic wall through bacteremia via the *vasa vasorum*, leading to inflammation, necrosis, and progressive structural destruction of the aortic wall. This process may ultimately evolve into an infectious (mycotic) aortic aneurysm and even catastrophic rupture [1, 2].

Although the most implicated pathogens in infectious aortitis are *Salmonella* spp., *Staphylococcus aureus* , and *Streptococcus* spp., the majority of infectious aortic aneurysms are caused by Gram‐positive organisms [3, 4] Gram‐negative pathogens account for a much smaller proportion, and *Escherichia coli* as the causative organism is exceedingly rare, often resulting in delayed diagnosis and substantial therapeutic challenges [2, 4, 5].

Previous studies have suggested that in patients with immunocompromised status, advanced age, diabetes mellitus, atherosclerosis, or pre‐existing aortic pathology, *E. coli* bacteremia—most commonly originating from the gastrointestinal or urinary tract—may disseminate to the aortic wall and trigger infectious aortitis or mycotic aneurysm formation [2, 4] The clinical course of this condition is highly variable, ranging from fulminant progression to an indolent, slowly evolving process [1, 4, 6] Regardless of the tempo of disease progression, infectious aortitis carries an inherently high risk of aneurysmal rupture and mortality [1, 4, 6].

In the pre‐antibiotic era, infectious aortitis was most frequently associated with bacterial endocarditis [1, 2] In contrast, in modern clinical practice, the predominant pathogenic mechanisms have shifted toward hematogenous spread during bacteremia, contiguous extension from adjacent infectious foci, or infections related to invasive medical procedures [1, 2] Although Gram‐negative organisms have increasingly been recognized as important pathogens in infectious aortic disease, *E. coli* has long been regarded as an incidental causative agent and has been reported primarily in isolated case reports [2, 6].

## 2 Case Presentation

A 76‐year‐old man with a medical history of coronary artery disease, ischemic stroke, chronic kidney disease, prior pulmonary embolism without regular follow‐up or medical control, and a previous episode of sepsis six months earlier was transferred from a local hospital with suspected rupture of an abdominal aortic aneurysm. He had experienced several days of progressive generalized weakness accompanied by nonspecific abdominal discomfort and increased sputum production. He initially sought medical attention at the local hospital because of generalized weakness and a temperature of 37.9°C on presentation. Follow‐up ultrasonography revealed focal aortic wall thickening with calcified spots over the epigastric region. No valvular vegetation was detected on echocardiographic examination. Subsequent abdominal computed tomography angiography (CTA) confirmed the presence of an abdominal aortic aneurysm, and he was therefore referred to our hospital for further management.

On arrival, he was hemodynamically stable, although mild hypotension and tachypnea were noted. Laboratory investigations revealed leukopenia (white blood cell count 0.96 × 10^3^/μL), anemia (hemoglobin 6.6 g/dL), thrombocytopenia (platelets 110 × 10^3^/μL), hyponatremia (127 mmol/L), hypocalcemia (6.3 mg/dL), and elevated serum lactate (2.4 mmol/L).

## 3 Differential Diagnosis, Investigations, and Treatment

**FIGURE 1:**
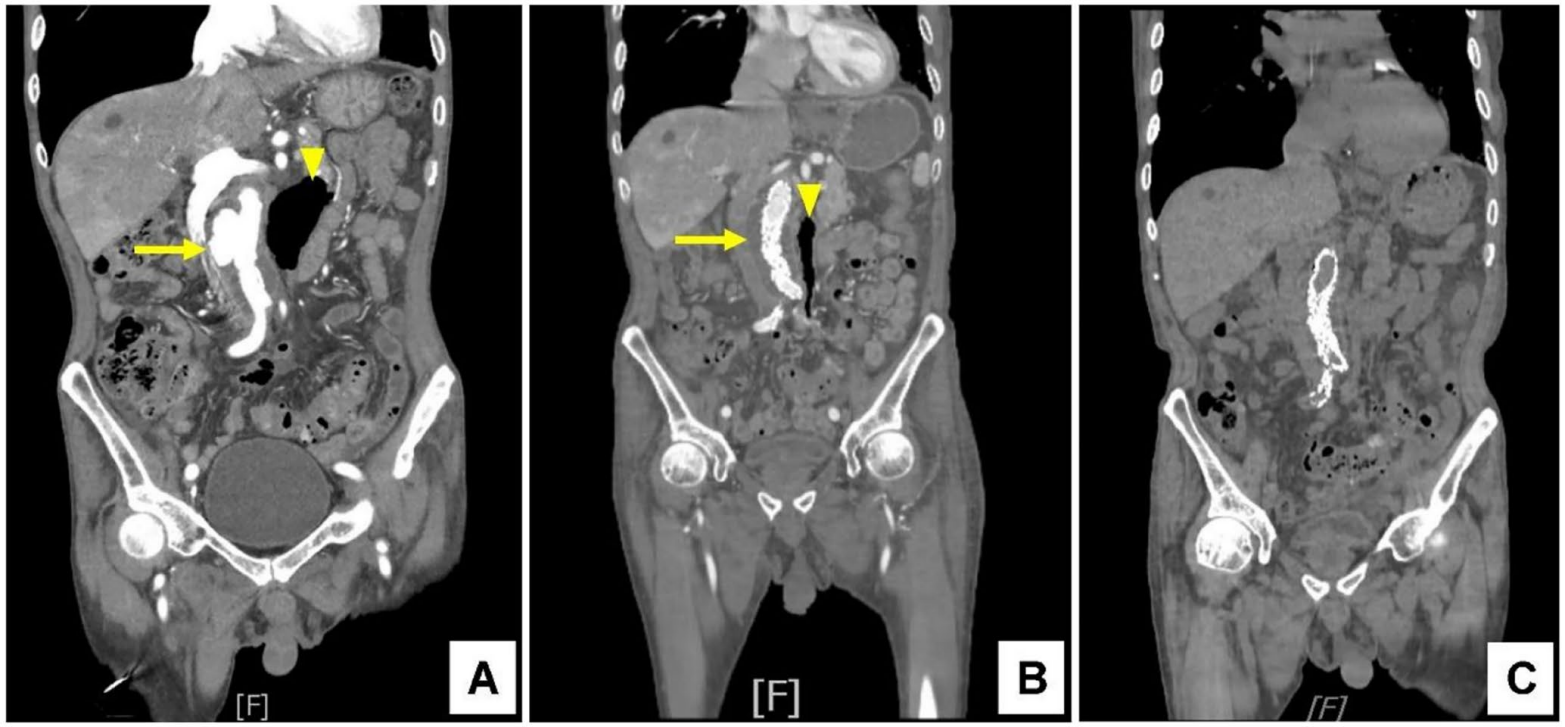
Serial contrast‐enhanced computed tomography angiography (CTA) of the abdomen. (A) Preoperative coronal CTA demonstrates an infrarenal abdominal aortic aneurysm (AAA) with an irregular aneurysmal contour (yellow arrow). Adjacent para‐aortic inflammatory changes with gas‐containing collections are noted (yellow arrowhead), consistent with infectious aortitis, likely originating from contiguous sigmoid diverticulitis. (B) Post‐percutaneous endovascular aneurysm repair (PEVAR) coronal CTA shows successful exclusion of the aneurysmal sac with transformation of the AAA into a thrombosed pseudo‐lumen (yellow arrow). Marked interval reduction of the adjacent diverticulitis‐associated gas cavity and para‐aortic inflammatory changes is observed (yellow arrowhead). (C) Follow‐up coronal CTA demonstrates favorable recovery, with stable stent graft position, no evidence of endoleak, and near‐complete resolution of periaortic inflammation and gas collections, indicating good post‐treatment response.

Given the concern for impending aneurysmal rupture, contrast‐enhanced CTA of the abdomen was performed. Imaging demonstrated an infrarenal eccentric saccular abdominal aortic aneurysm measuring approximately 3.9 cm with peripheral mural thrombus and associated diverticulitis (Figure 1A). No definite aneurysmal rupture was identified. Additional findings included bilateral renal pelvis dilatation with multiple renal cysts, transient hepatic attenuation difference with benign‐appearing, non‐enhancing hepatic cysts, and infiltrative changes in the left lower lung lobe suggestive of a possible inflammatory or infectious process. Blood cultures obtained upon admission subsequently yielded *E. coli* .

On arrival, the patient exhibited relative hypotension (blood pressure 96/66 mmHg), raising concern for potential hemodynamic deterioration. Given his advanced age, multiple comorbidities, ongoing bacteremia, and borderline hemodynamic status, an endovascular approach was considered to minimize operative stress and avoid the risks associated with open surgical repair.

The patient underwent emergent percutaneous endovascular aneurysm repair (PEVAR) under intravenous general anesthesia with high‐flow nasal cannula oxygen support. Percutaneous access was achieved via the left common femoral artery using a 7‐Fr sheath, with pre‐closure performed using two ProGlide devices. A pigtail catheter was used for angiographic localization of the rupture site. After guidewire exchange to a stiff wire, a modular three‐piece Endurant II stent‐graft system was initially deployed in an attempt to exclude the infrarenal aneurysm. However, intraoperative angiography revealed that the aneurysmal outpouching extended to the level of the aortic bifurcation, resulting in inadequate distal sealing with the standard bifurcated configuration. To achieve secure exclusion of the lesion and reconstruct the aortic bifurcation, a covered endovascular reconstruction of the aortic bifurcation (CERAB) technique was subsequently performed. This involved placement of a covered aortic stent extending to the bifurcation, followed by deployment of two covered iliac stents into the bilateral common iliac arteries (right: 13 mm; left: 10 mm), creating a neo‐bifurcation configuration.

Completion angiography confirmed complete exclusion of the aneurysm sac without evidence of endoleak and preserved visceral artery perfusion. Final hemostasis was achieved using additional ProGlide devices, and the femoral access site was closed. Postoperatively, the patient was admitted to the intensive care unit and empirically treated with intravenous metronidazole, ceftriaxone, and vancomycin. Antimicrobial therapy was later adjusted based on susceptibility testing.

## 4 Outcome and Follow‐Up

The patient demonstrated steady clinical improvement and was transferred out of the intensive care unit on hospital Day 6. Vital signs remained stable, and no further febrile episodes were observed. Follow‐up imaging showed no evidence of endoleak or aneurysm progression (Figure 1B and Figure 1C). He was discharged in stable condition on hospital Day 34 with outpatient follow‐up arranged.

## 5 Discussion

*E. coli‐* associated mycotic aortic aneurysms predominantly occur in elderly male patients and are frequently accompanied by urinary tract infections, gastrointestinal infections, or persistent or recurrent bacteremia [2]. Since the early 2000s, the rising prevalence of multidrug‐resistant *E. coli* strains, including extended‐spectrum β‐lactamase (ESBL)‐producing isolates, has drawn increasing attention to their role in aortic infections, particularly among patients with recurrent bacteremia or suboptimal responses to antibiotic therapy [1, 5].

Although *E. coli* ‐related infectious aortitis remains rare, the number of reported cases has increased over time. Most patients require CT or CTA for early and accurate diagnosis. Typical imaging findings include aortic wall thickening, periaortic soft‐tissue inflammation, fluid or gas collections, and pseudoaneurysm formation [1, 2, 7]. Given the high mortality associated with infected aortic aneurysms, prompt initiation of antimicrobial therapy is essential once the diagnosis is suspected [1, 2, 4]. Infected aortic aneurysm caused by Escherichia coli requires early administration of broad‐spectrum intravenous antibiotics [2, 6]. Several reports emphasize that empiric antimicrobial therapy should be started preoperatively in the presence of bacteremia or radiologic evidence of periaortic inflammation to reduce the risk of rupture and systemic dissemination [2, 6]. After surgical intervention and microbiological confirmation, antimicrobial therapy should be tailored according to culture susceptibility results. Regarding treatment duration, most studies recommend at least six weeks of intravenous antibiotic therapy following surgical debridement [4, 6]. Prolonged therapy is advocated due to poor antibiotic penetration into the infected aortic wall, the risk of residual infection after vascular reconstruction, and the potential for graft infection [2, 5]. In selected high‐risk patients, long‐term suppressive oral therapy may be considered, particularly in immunocompromised hosts or when complete eradication of infection is uncertain [1].

Surgical management of aortic disease encompasses a variety of approaches, selected according to disease severity and anatomical extent [3]. Over the past several decades, treatment strategies have evolved substantially—from conventional open surgical techniques, such as aneurysm resection and bypass grafting, to less invasive endovascular interventions, including endovascular aneurysm repair (EVAR) and thoracic endovascular aortic repair (TEVAR) [2, 8, 9].

Furthermore, recent case reports have suggested a potential association between infectious aortic aneurysms and gastrointestinal lesions, such as diverticulitis, analogous to the well‐established relationship between *Clostridium septicum* infection and colonic pathology [10]. These findings underscore the importance of concurrently evaluating potential gastrointestinal sources of infection when diagnosing *E. coli* ‐associated aortic infections [1, 10].

## Author Contributions

**Ching‐Chou Pai:** conceptualization, data curation, investigation, resources, writing – original draft. **Ya‐Ting Chuang:** data curation, investigation, project administration. **Hua‐Yu Lin:** data curation, investigation. **Tao‐An Chen:** conceptualization, methodology, writing – original draft, writing – review and editing.

## Funding

The authors have nothing to report.

## Ethics Statement

The authors have nothing to report.

## Consent

Written informed consent was obtained from the patient(s) for their anonymized information to be published in this article.

## Conflicts of Interest

The authors declare no conflicts of interest.

## Data Availability

Data sharing not applicable to this article as no datasets were generated or analysed during the current study.
